# Proximal Fibulectomy for Giant Cell Tumours: What Works!

**DOI:** 10.1007/s43465-024-01231-2

**Published:** 2024-09-03

**Authors:** Ashwin Prajapati, Harsha S. S. Tadala, Ashish Gulia, Ajay Puri

**Affiliations:** 1 Department of Surgical Oncology, Homi Bhabha National Institute, Tata Memorial Hospital, Parel, Mumbai, Maharashtra India; 2 Present Address: Department of Surgical Oncology, Marengo CIMS Cancer Centre, Off Science City Road, Sola, Ahmedabad, Gujarat India; 3 Present Address: Department of Surgical Oncology, Homi Bhabha Cancer Hospital and Research Centre, Tata Memorial Centre, Visakhapatnam, Andhra Pradesh India; 4 Present Address: Department of Surgical Oncology, Homi Bhabha Cancer Hospital & Research Centre, Tata Memorial Centre, Mohali, New Chandigarh, Punjab India

**Keywords:** Proximal fibulectomy, Lateral collateral ligament, Common peroneal nerve, Giant cell tumor

## Abstract

**Background:**

Giant cell tumor of bone (GCTB) is the most common primary tumor of proximal fibula. Because of its close proximity to vascular structures, common peroneal nerve (CPN) and attachment of lateral collateral ligament (LCL), proximal fibulectomy poses unique challenges. We analyzed oncological and functional outcome of patients who underwent proximal fibulectomy for GCTB of proximal fibula.

**Material and methods:**

Between January 2006 and December 2020, 23 patients underwent proximal fibulectomy for GCTB of proximal fibula, four were recurrent tumors. Mean resection length was 9 cm (5 to 15 cm). The LCL and biceps tendon were not reconstructed in 22 cases. The common peroneal nerve was sacrificed in seven patients including three recurrent cases. Functional status was assessed using the Musculoskeletal Tumour Society (MSTS) scoring system.

**Results:**

There were two vascular complications and one infection. With 4 patients lost to follow up, mean follow up was 90 months (12 to 197). No patient had local or distant recurrence. Mean MSTS score was 26 (21 to 30). Eleven of 23 patients (48%) had loss of common peroneal nerve function with poorer functional outcome. No patient had symptoms suggestive of knee instability.

**Conclusion:**

Proximal fibulectomy is oncologically safe. Reconstruction of the LCL attachment is not mandatory and patients do not have symptomatic knee instability. Functional outcomes are compromised after sacrifice of common peroneal nerve and may be potentially improved with tendon transfers at index surgery.

## Introduction

Primary bone tumours of the proximal fibula are relatively uncommon and account for only 4% of all the primary bone tumours [1]. Giant cell tumour of bone (GCTB) is commoner than other malignant or benign tumors at this location [2]. Being an expendable bone, resection rather than curettage is the mainstay of treatment even for benign bone tumors at this location [3]. Though it is considered an expendable bone, proximal fibulectomy can result in lateral instability of the knee joint. Multiple musculotendinous attachments and proximity to the common peroneal nerve (CPN) and the vascular trifurcation makes surgical resection of proximal fibular tumours challenging.

We analysed our results of surgical resection of giant cell tumor of proximal fibula with respect to local recurrence, CPN dysfunction and functional outcomes.

## Materials and Methods

After approval by the institute’s ethics committee, we retrospectively reviewed our prospectively maintained surgical database of operated patients. Over a period of 15 years (January 2006 and December 2020), a total of 26 patients with proximal fibula GCTB underwent surgery. Three patients operated for soft tissue recurrence after proximal fibulectomy done outside were excluded. Of the remaining 23 patients, four were recurrent tumors who had been treated with curettage elsewhere. There were 14 males and 9 females with a mean age of 24 years (range 16 to 46). Their medical records, imaging, functional and current oncological status were reviewed.

**Fig. 1:**
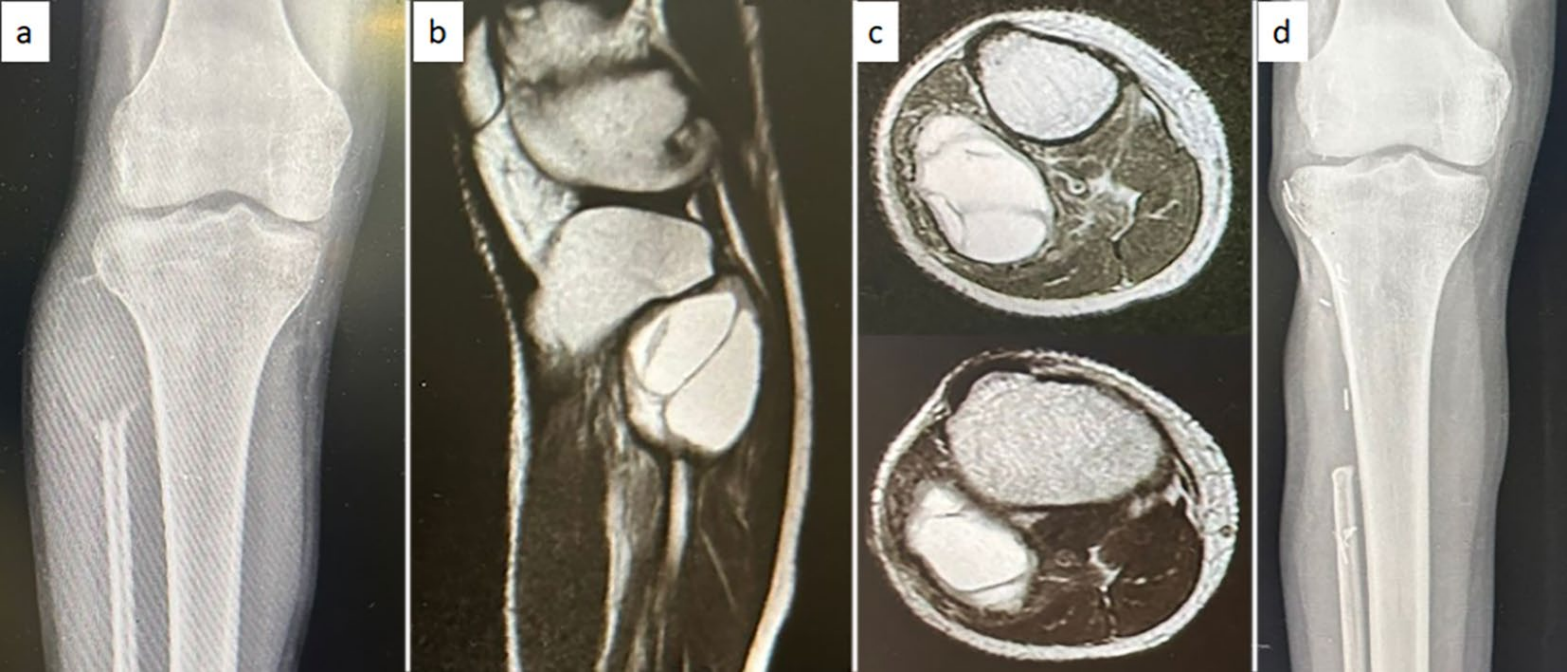
Radiological images of proximal fibula GCT; **a** pre operative radiograph showing expansile lytic lesion, **b** & **c** MRI images in sagittal and axial plane showing secondary ABC (Aneurysmal bone cyst) component, **d** post operative radiograph after proximal fibulectomy

During initial evaluation all patients were imaged with a plain radiograph in two planes and magnetic resonance imaging of the entire fibula (Fig. 1). Twenty patients had Campanacci grade 3 tumor and 3 patients had Campanacci grade 2 tumor. All patients were operated only after confirming the histopathology either by a core needle biopsy at our institute or a review of a biopsy done elsewhere. Staging investigations included a plain radiograph of the chest. All patients were non-metastatic at presentation.

Neoadjuvant denosumab was given to two patients pre-operatively to facilitate resection (after dental check-up and serum calcium levels). The decision to give denosumab was based on the surgeon’s perception for potential complications (tumor spillage/injury to neurovascular structures) [4]. Dosage schedule was: Inj Denosumab 120 mg, SC (subcutaneous), on 0, 7, 14, 28 days and reassessedbetween 3 to 4 week from the last dose. If response was good (increased calcification/ossification of tumor on radiograph/ decrease in size on MRI / clinical response in the form of decreased swelling and pain) we proceeded for surgery at the earliest.

### Surgical Technique

- Best to use a tourniquet and operate in the floppy lateral position with a lateral approach.
- En bloc excision of the proximal fibula with adequate oncologic margins (as planned based on pre operative MRI) in all patients.
- Decision to preserve / sacrifice nerve based on preoperative evaluation of MRI, an unsuccessful intraoperative attempt to dissect an involved nerve can lead to inadvertent contamination.
- Nerve is identified at the level of knee joint with just posterior to biceps tendon and traced distally. Keeping knee joint in flexion makes identification of nerve proximally easier. Some of the proximal muscular branches of deep peroneal nerve had to be sacrificed to free the nerve from the tumor.
- Origin of soleus from fibula is erased to expose the trifurcation of the vessels. Proximal ligation of peroneal vessels (which are commonly sacrificed) is done only after completely identifying posterior tibial vessles along their course.
- Fibula osteotomy is done as planned and the specimen is rotated externally to facilitate further dissection.
- Biceps and LCL insertion is erased from proximal fibula. Proximally, tibio-fibular joint cartilage provides a good margin and if the tibio-fibular joint is involved a sliver of proximal tibia is excised with the specimen.
- Biceps and LCL are sutured to surrounding soft tissue and the wound is closed in layers over a negative suction drain.

Mean resection length was 9 cm (5 to 15 cm). In one case a sliver of tibia at the tibio-fibular joint was excised along with the proximal fibula in order to ensure adequate margins. In one case a prolene mesh (Prolene TM Mesh, Johnson & Johnson, Ethicon division, Aurangabad, India) was used to augment joint stability on the lateral aspect of the knee joint.

The CPN was sacrificed in 7 patients (30%) as it was involved by the tumour and any attempt to retain it would have resulted in extensive tumor contamination. Three of the four patients with a recurrent tumor underwent sacrifice of the nerve, while 4 of 19 with a primary tumor needed sacrifice of the nerve.

### Rehabilitation

Patients were allowed full weight bearing mobilization in the immediate postoperative period with a long leg knee brace. Patients having foot drop were given a foot drop splint. After 3 weeks the long knee brace was discontinued and knee range of motion exercise started. Quadriceps, ankle flexor and extensor muscle strengthening exercises were encouraged throughout the rehabilitation period.

Patients were asked to follow up every 6 months for the first 5 years and yearly there after till 10 years. At each follow-up they were assessed clinically and radiologically for oncological and functional outcomes. Local radiographs were done during each follow up visit and a chest radiograph every yearly. Patients were not specifically examined for knee joint stability. Functional status at last follow up was assessed using the Musculoskeletal Tumour Society (MSTS) scoring system [5]. MSTS score for lower limb is based on six factors i.e., pain, functional activity, emotional acceptance, use of external support, walking ability and gait. Each factor is given a value 0 to 5, the MSTS score is a sum of the value of these six factors. The maximum value being 30, a higher score denotes better function.

## Results

Three patients (13%) had complications. One patient had a posterior tibial artery injury, as the tumour was large and adherent to the vessel. This was repaired intraoperatively and the patient had an uneventful recovery. One patient who had a recurrent tumor had popliteal artery thrombosis in the immediate post-operative period and was managed conservatively with anti-coagulants. Post operative infection occurred in the one patient in whom mesh augmentation was done. This settled after surgery for removal of the mesh.

Three of 23 (13%) cases had positive margins, 2 soft tissue margins and one bone margin. Four of 23 patients did not follow up after surgery. In the remaining 19 patients mean follow up was 90 months (range 12 to 197). None of the patients developed local recurrence or distant metastasis. All three patients with positive margins have not had recurrence with more than 6 years follow up.

Of the 16 patients in whom the CPN was preserved at surgery, six developed post-operative palsy. Two of these subsequently recovered. Thus 4 of 16 patients (25%) in whom the nerve was preserved had palsy while 11 of the total 23 patients (48%) had loss of CPN function (Table 1).

**Table 1:** xxx

Sr no	Age	Sex	Intraosseous extent	Follow-up	CPN sacrificed	Foot drop
1	41	M	5	141	No	No
2	27	M	10	174	No	No
3	33	M	9	104	No	Yes
4	21	F	8	78	Yes	Yes
5	18	M	11	77	Yes	Yes
6	31	F	11	124	Yes	Yes
7	17	F	13	86	Yes	Yes
8	27	F	9	85	No	No
9	21	F	14	197	No	No
10	21	M	5	102	No	No
11	16	F	6	91	No	No
12	21	M	9	87	No	Yes
13	21	F	8	LFU	No	No
14	28	M	8	LFU	No	No
15	46	M	8	31	No	No
16	19	F	4	137	No	No
17	21	M	10	58	Yes	Yes
18	18	M	7	49	Yes	Yes
19	21	M	4	44	No	No
20	25	F	9	12	Yes	Yes
21	19	M	11	LFU	No	No
22	20	M	6	40	No	Yes
23	28	M	6	LFU	No	Yes

*Sr no:* Serial number, *cm:* centimetre, *CPN:* Common peroneal nerve, *M:* Male, *F:* Female, *LFU:* Lost to follow up

The MSTS score for 19 patients recorded at their last follow up visit ranged from 21 to 30 with a mean of 26. Functional scores in patients with loss of common peroneal function ranged from 21 to 25 (mean 23) and in the patients with a functional nerve ranged from 26 to 30 (mean 28). While none of the patients was specifically questioned regarding knee instability, no patient had symptoms suggestive of instability.

## Discussion

The fibula is a relatively uncommon site for bone tumours. Being an expendable bone, both benign and malignant tumors are usually treated with en bloc resection as curettage in benign tumors has a high rate of local recurrence [3]. The close proximity of neuro vascular structures in this anatomic location makes resection challenging with a high incidence of post-operative sequalae.

While some authors recommend saving the CPN in benign tumors, to salvage or sacrifice the CPN is best decided on a case to case basis depending on its involvement by the tumor. We had to sacrifice the CPN in seven patients as it was completely encased. Our higher rate of sacrifice of the CPN compared to other case series [2, 3] could be a reflection of larger tumor size and recurrent tumors. The rate of sacrifice was 75% in recurrent tumors and 21% in primary tumors. Even in the 16 patients in whom the nerve was preserved, 6 still developed post-operative nerve palsy. Only 2 of these subsequently recovered. Thus, 48% of our patients had permanent nerve palsy after proximal fibula resections highlighting the importance of counseling patients regarding this pre-operatively even in benign tumors.

Proximity of the vascular trifurcation at this anatomic site can result in vascular complications which have been reported in other case series as well [2]. We had two cases of vascular complications, both were adequately treated with timely detection and intervention and recovered without any long-term consequences. Meticulous post-operative monitoring and prompt intervention ensure minimal long term sequalae.

Three of our patients had positive resection margins. Surprisingly we had no local recurrence even in patients with positive margins. Abdel et al. [3] reported 11% recurrence rate following resection of proximal fibula GCT when compared with curettage (66%). Though the desire to gain adequate oncologic margins has to be moderated by the functional morbidity especially in benign tumors, our lower rate of recurrence could be a reflection of our willingness to sacrifice the nerve (when involved by tumor) and the decision to include a sliver of tibia at the tibio fibular joint when required to ensure adequate margins.

Post-operative function is mainly influenced by the status of the CPN. MSTS scores were lower in patients with nerve palsy ranging from 21 to 25 compared with patients with intact nerve function who had MSTS score ranging from 26 to 30.

For the patients in this series we did not practice immediate tendon transfers after sacrifice of the CPN. We preferred to wait for 2 years till the chance of local recurrence was minimal before recommending tendon transfers if the patient desired it. Over the past few years, encouraged by our excellent local control we now discuss with and offer patients the option of tendon transfers at index surgery to compensate for sacrifice of the CPN and have better functional outcomes [2, 3].

Though the lateral collateral ligament (LCL) is theoretically a stabilizer of the knee joint, the need for its reconstruction/repair after proximal fibula resections is debatable [2, 3, 6]. In Inatani’s series 4 of 12 patients did not undergo reconstruction of LCL and none of them reported instability. Lateral collateral reconstruction yielded better results in a series of 46 patients by Kundu [2] Abdel [3] too in a study of 121 patients, recommended LCL repair for better long-term outcomes. We did not reconstruct the LCL or biceps tendon. The lateral soft tissues were carefully apposed and sutured. The one case in which a prolene mesh was used on the lateral aspect of knee joint to augment joint stability had infection that necessitated removal of the mesh. None of our patients had symptoms suggestive of instability. Possibly, the other stabilizers of the knee like the joint capsule, cruciate ligaments, an intact iliotibial band aided by the fibrosis as a result of surgery provided adequate stability.

## Conclusion

GCTB of proximal fibula are rare tumours. While resection ensures excellent local control, patients need to be counseled pre-operatively regarding the potential complications. Reconstruction to compensate for loss of the LCL attachment is not mandatory and patients do not complain of knee instability. Functional outcomes are determined by presence of a functional CPN and may be potentially improved with tendon transfers at index surgery in case the nerve is sacrificed.
